# Artificial intelligence electrocardiography for left ventricular systolic dysfunction demonstrates preserved performance across demographic training imbalances

**DOI:** 10.1093/ehjdh/ztag080

**Published:** 2026-05-28

**Authors:** P Nelson Hsieh, Parth Agrawal, Aman Alok, Sathis Kumar, Charu Ramanathan, Venkatesh L Murthy, Niraj Varma, Venkat Nagarajan, Andrew P Ambrosy, Mattheus Ramsis, Antonis A Armoundas, Jagmeet P Singh

**Affiliations:** Division of Cardiology, Massachusetts General Hospital, Harvard Medical School, 55 Fruit St, Boston, MA, USA; Carelog Inc, CA, USA; Carelog Inc, CA, USA; Carelog Inc, CA, USA; Carelog Inc, CA, USA; Division of Cardiovascular Medicine, University of Michigan, Ann Arbor, MI, USA; Department of Cardiovascular Medicine, Cleveland Clinic, Cleveland, OH, USA; Department of Cardiology, Kokilaben Dhirubhai Ambani Hospital, Mumbai, India; Department of Cardiology, Kaiser Permanente SanFrancisco Medical Center, San Francisco, CA, USA; Division of Research, Kaiser Permanente Northern California, Pleasanton, CA, USA; Division of Cardiovascular Medicine, University of California, San Diego, CA, USA; Cardiovascular Research Center, Massachusetts General Hospital, Boston, MA, USA; Broad Institute, Massachusetts Institute of Technology, Cambridge, MA, USA; Division of Cardiology, Massachusetts General Hospital, Harvard Medical School, 55 Fruit St, Boston, MA, USA

**Keywords:** Foundational model, AI-ECG, Heart failure, LV systolic dysfunction, Demographics, Artificial intelligence, ECG

## Abstract

**Aims:**

Artificial intelligence (AI)-enabled electrocardiograms (AI-ECG) can detect left ventricular systolic dysfunction (LVSD), but demographic imbalance in training datasets may introduce bias. Foundational models, pretrained on large and diverse datasets, may mitigate such concerns. We aimed to assess the impact of demographic composition in training datasets on the performance of an ECG Foundational Model (ECGFM) for diagnosing LVSD.

**Methods and results:**

We developed an ECG foundational model (ECGFM) using transformer architecture and self-supervised pretraining on 983 200 ECGs. Using 44 815 paired ECG–echocardiogram datasets, we trained the model under three biased scenarios: (1) sex-skewed (male-only or female-only), (2) race-skewed (White-only or non-White), and (3) balanced. Models were evaluated on a test cohort consisting of 4663 male patients (52%) and 4300 female patients (48%) for the sex configuration and 4440 (49.5%) White, 558 (6.2%) Black, 925 Asian (10.3%), and 3040 other (33.9%) patients, for the race-based configuration using area under the receiver operating characteristic curve (AUROC). The ECGFM demonstrated consistent performance across all demographic configurations. Training on male-only or female-only cohorts yielded comparable AUROC scores of 0.85–0.90 for both sexes in the test set in predicting LVSD. Similarly, training on White-only or non-White cohorts resulted in robust AUROC scores (≥0.90) across all racial groups, including Asian, Black, Hispanic/Latino, and American Indian/Native Alaskan subgroups. Balanced and imbalanced training produced comparable accuracy, sensitivity, and specificity. The performance of the model was externally tested in EchoNext, revealing AUROC scores 0.823–0.917 for sex and 0.822–0.917 for race.

**Conclusion:**

Our transformer-based ECG foundational model pretrained using self-supervised learning demonstrated preserved diagnostic accuracy for LVSD across diverse demographic groups, even when trained on demographically imbalanced datasets.

## Graphical Abstract

**Graphical Abstract:**
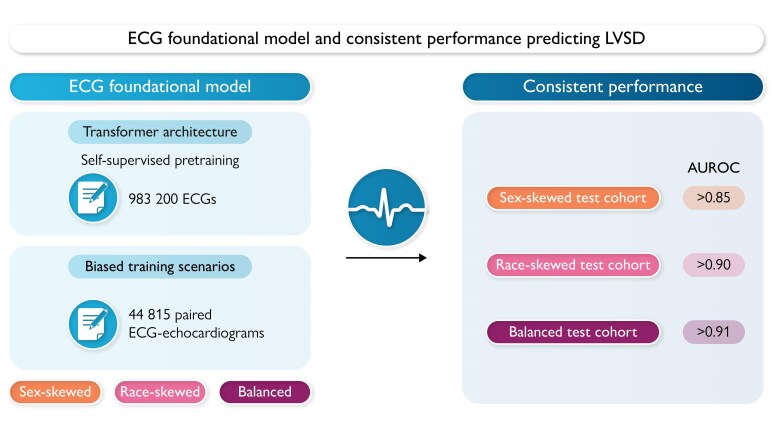
An ECG foundational model for predicting LVSD demonstrates robust generalization across sex and racial groups

## Introduction

Heart failure represents a global epidemic affecting more than 64 million people, and its most morbid forms are often associated with reduced left ventricular ejection fraction (LVEF).^1,2^ Prior to the development of clinical heart failure, early detection of asymptomatic LV systolic dysfunction (LVSD) can facilitate intervention with evidence-based therapies to reduce patient symptoms and improve survival.^3^ Transthoracic echocardiography (TTE) is the gold standard for diagnosis of low LVEF but is prohibitively expensive to deploy for population-wide screening and is unavailable in resource-constrained settings.^3^ In response, artificial intelligence–based electrocardiography (AI-ECG) has been developed to accurately detect systolic and diastolic dysfunction, identify cardiomyopathies, and enhance clinical risk stratification, often before it rises to the threshold of human attention.^4–8^ At least one approach has demonstrated cost-effectiveness of an AI-aided strategy for targeted large population screening for LVSD in the outpatient clinic setting.^9^

A central challenge to any AI-enabled ECG development is demographic skew in training datasets.^10,11^ The impact of demographic composition during development (training and validation) on the performance of ECG-based AI models for predicting LVSD remains poorly understood; however, sex and racial differences in cardiac physiology and ECG characteristics have been well-documented.^12,13^ These are easily perpetuated in AI prediction models and reinforce existing inequities, reduce generalizability, and decrease performance.^12,14–16^ This can occur even when algorithms are explicitly designed to exclude race in their calculations.^17^ Attempts to overcome this limitation centre around the accumulation of ever-expanding data sets, which exhibit less demographic imbalance.

Foundational models, defined as self-supervised AI models whose number of parameters usually vastly exceeds previous machine learning algorithms, and are trained on datasets of many orders of magnitude greater in scale and diversity, hold promise in addressing demographic variability in the training data.^18,19^ ECG foundational models have yet to achieve the performance of large language models in diagnosing LVSD, yet efforts are underway to harness their generalizability to develop demographic-agnostic AI-ECG models, which perform well across clinical settings.^20,21^

In this study, we present the development and validation of an ECG Foundational Model (ECGFM) to diagnose LVSD. We evaluated its performance after training under different configurations for sex and race and demonstrated equal and broad discriminatory capacity in all subgroups of race and sex.

## Methods

### Study design and setting

In this retrospective observational study using paired ECG and TTE datasets, we evaluated the impact of demographics on the performance of a foundational AI-ECG model for diagnosing LVSD, determined by LVEF (<40%). We used LVEF <40% to align with guideline definitions of heart failure with reduced EF and ensure clinical relevance of screening.^22^ This study utilized de-identified clinical data provided by Dandelion Health. All data were de-identified in accordance with the HIPAA privacy rule via expert determination and contained no individually identifiable health information. As such, this research (under the definition of 45 CFR 46.102) was determined to be exempt from the Institutional Review Board. The data underlying this article are available in PhysioNet, at https://doi.org/10.13026/3ykd-bf14, or were provided by Dandelion Health under license/by permission. Data will be shared on request to the corresponding author with permission from Dandelion Health.

### Data sources and population

**Figure 1:**
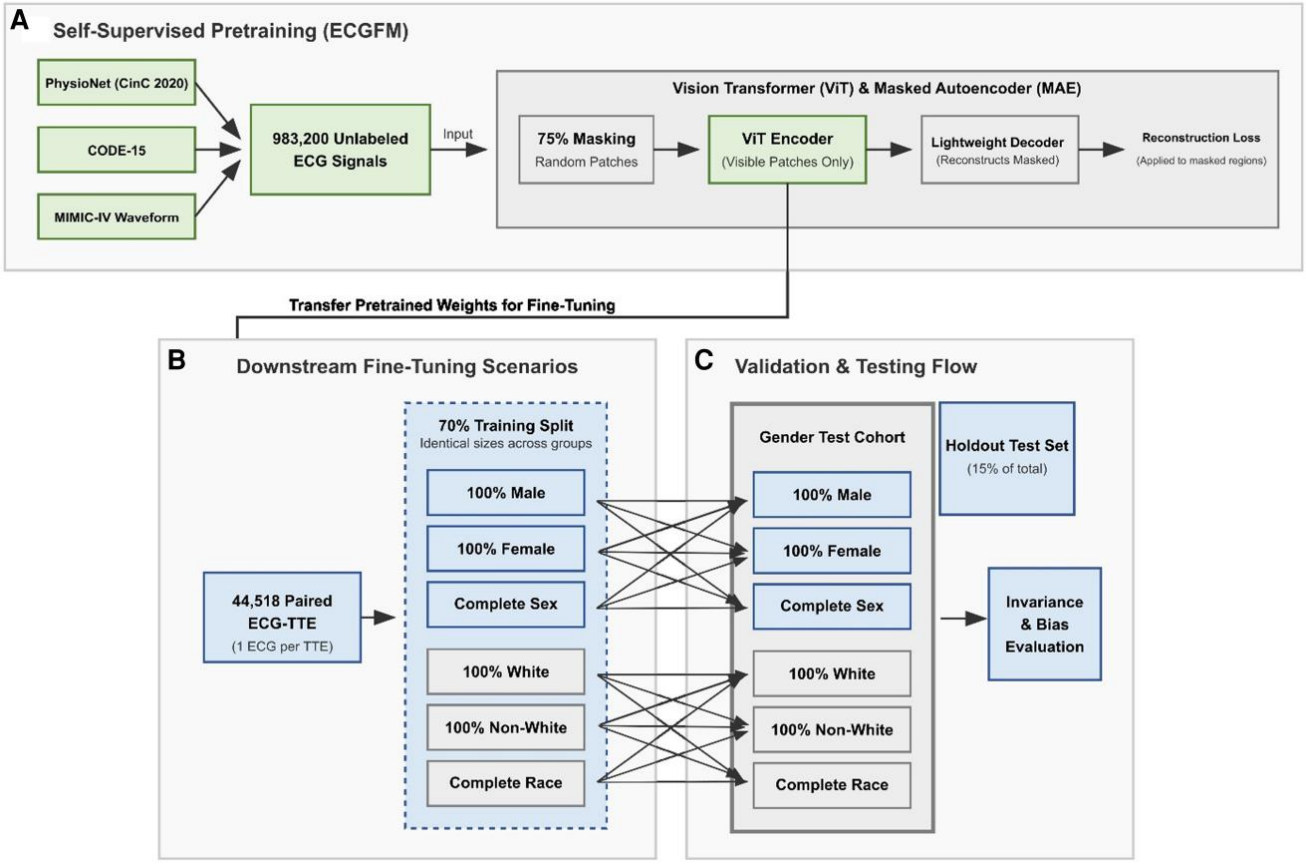
Schematic of dataset creation and analysis.

A retrospective review of 44 815 paired ECGs and TTE datasets was performed. The data were collected between August 2019 and October 2023. These data originate from three large US health systems: Sharp Healthcare (California), Sanford Health (Dakotas/Midwest), and Texas Health Resources (Texas), providing broad geographic coverage across the Western, Midwestern, and Southern United States. This included both inpatient and outpatient settings where patients received both an ECG and a TTE within 30 days. Only one unique ECG-echo pair was included per patient, and there was no overlap between the pretraining corpora and the downstream data. The inclusion criteria were (1) adults aged >18, and (2) an ECG and TTE performed within a maximum interval of 30 days to ensure temporal correlation. Exclusion criteria included: (1) patients with missing or incomplete TTE data and (2) patients with paced rhythms. **(***Figure 1***)**.

### Data preprocessing

ECG signals were pre-processed using standardized noise filtering to remove baseline wander and high-frequency artefacts and normalization of signal amplitude and duration to ensure consistency across samples. Signal duration was standardized to 10-s 12-lead ECG recordings. Signals were sampled at 250 Hz. The ECG signal (12 × 2500, i.e. 10 s of 12 leads ECG signal sampled at 250 Hz) was divided into patches of size 1 × 50, corresponding to 1/5 s of signal. This resulted in 12 × 50 = 600 patches.

### ECG foundational model overview

To comprehensively evaluate the impact of demographic composition on the performance of the ECG Foundational Model (ECGFM) for diagnosing LVSD, we designed an experimental framework. This framework included three distinct training scenarios for both sex (three datasets composed of 100% male, 100% female, and 52% male/48% female patients) and race (three datasets composed of 100% White, 100% non-White, and 49.5% White/50.5% non-White patients), with testing conducted across all relevant demographic subgroups. The goal was to assess whether training data composition influenced the model’s generalizability across populations with diverse demographics. For each scenario, we ensured that the total number of ECGs used for training was identical across all scenarios (e.g. Male-only vs. Female-only vs. Complete Sex Dataset; White-only vs. Non-White vs. Complete Racial Dataset). This eliminated confounding effects due to differences in dataset size.

In addition to the training datasets (70%), the remainder of the data was split into validation (15%), and testing (15%) cohorts using stratified sampling to ensure balanced representation across demographic groups. Cross-validation was performed during training to optimize hyperparameters such as learning rate, batch size, and regularization parameters. No ECGs from the same patient were used in training/validation and testing, and only one ECG was paired to an individual TTE.

Testing data sets for the sex and race-based configurations were demographically balanced. In the testing data sets, there were 4663 male patients (52%) and 4300 female patients (48%) for the sex configuration, and 4440 (49.5%) White, 558 (6.2%) Black, 925 Asian (10.3%), and 3040 other (33.9%) patients, for the race-based configuration. This subgroup analysis enabled a detailed evaluation of whether models trained on specific demographic groups exhibited biases or performance disparities when tested on other groups. Stratified sampling was employed during dataset splitting to maintain proportional representation (e.g. LVEF <40%) across training, validation, and test sets. The validation and test sets were kept constant across all configurations to provide a consistent benchmark for comparing model performance. Stratified five-fold Monte Carlo cross-validation was performed during training to ensure robust evaluation of each configuration.

External validation was performed using the publicly available ECG-ECHO paired EchoNext dataset collected at Columbia University Irving Medical Center comprised of patient records who received both within a 1-year interval between 2008 and 2022, resulting in a total dataset of 54 636.^23,24^

### Model architecture and training

The ECGFM was pretrained using a Vision Transformer (ViT) model and Masked Autoencoder based self-supervised learning framework on 983 200 ECG signals, drawing from a large-scale dataset comprising three prominent sources: the PhysioNet/Computing in Cardiology Challenge 2020 dataset, the CODE-15 dataset, and the MIMIC-IV Waveform Database. These datasets collectively provided a diverse and extensive pool of ECG signals, enabling the model to learn robust representations of ECG features through self-supervised learning.

The masking strategy is a self-supervised training method to make use of abundant unlabelled ECG signals and help the model learn characteristics/representations of ECG signals.^25^ This methodology has proven effective in the computer vision domain as well as for ECG signals, and it has previously been shown that a model pretrained with masked autoencoding gives better results when fine-tuned for the classification task using a labelled ECG dataset.^26^ The masking strategy utilized a 75% masking ratio to randomly select patches. Only unmasked patches were passed through the encoder to reduce computational complexity while retaining essential information. The decoder reconstructed the masked patches using visible patch embeddings and mask tokens. The ViT encoder processed only the visible patches along with the positional encodings, leveraging self-attention mechanisms to capture temporal and spatial dependencies within the ECG signal. A lightweight decoder reconstructed the masked patches. The asymmetric design minimized computational overhead during pretraining, as the decoder was only used for reconstruction and not during fine-tuning. Reconstruction loss was computed as the mean absolute error between the original and reconstructed masked patches. This loss was applied exclusively to the masked regions, encouraging the model to learn meaningful latent representations from incomplete data. Cross-validation was performed during training to optimize hyperparameters such as learning rate, batch size, and regularization parameters.

### Model outcomes and statistical analysis

Model performance was assessed using area under the curve of the receiver operating characteristic (AUROC) as the primary measure with 95% confidence intervals (CIs) via bootstrapping, as well as sensitivity and specificity for true positive/negative rates and precision-recall curves for unbalanced datasets. The dataset was split into subsets using stratified sampling to ensure balanced representation across demographic groups.

## Results

A total of 1 366 141 ECGs and 165 924 TTEs were collected from 493 426 and 99 876 unique patients, respectively, between August 2019 and October 2023 and included in the study. After data pre-processing, a cohort of 56 960 unique ECG-echo pairs was identified, with 44 815 having EF values. Baseline patient characteristics of the study population are summarized in ***Table 1***.

**Table 1:** Baseline characteristics of the study population

Demographics and vitals	Mean ± SD	Median [interquartile range]
White*	49.9	
Male sex*	52.0	
Age, years	67 ± 16	68 [57–79]
Heart rate, beats/min	86 ± 24	82 [69–99]
**Comorbidities**	**Percentage**	—
Hx of heart failure	39.4	
Mean LVEF	58.1 ± 13.2	61.0 [53.0–67.0]
Patients with LVEF <40%	10.4	
Atrial fibrillation	32.0	
Coronary Revascularization	21.0	
Myocardial infarction	19.4	
Ventricular tachycardia	10.9	
Ventricular fibrillation	10.3	
Wide QRS complex	3.7	
Left bundle branch block	2.84	

**Figure 2:**
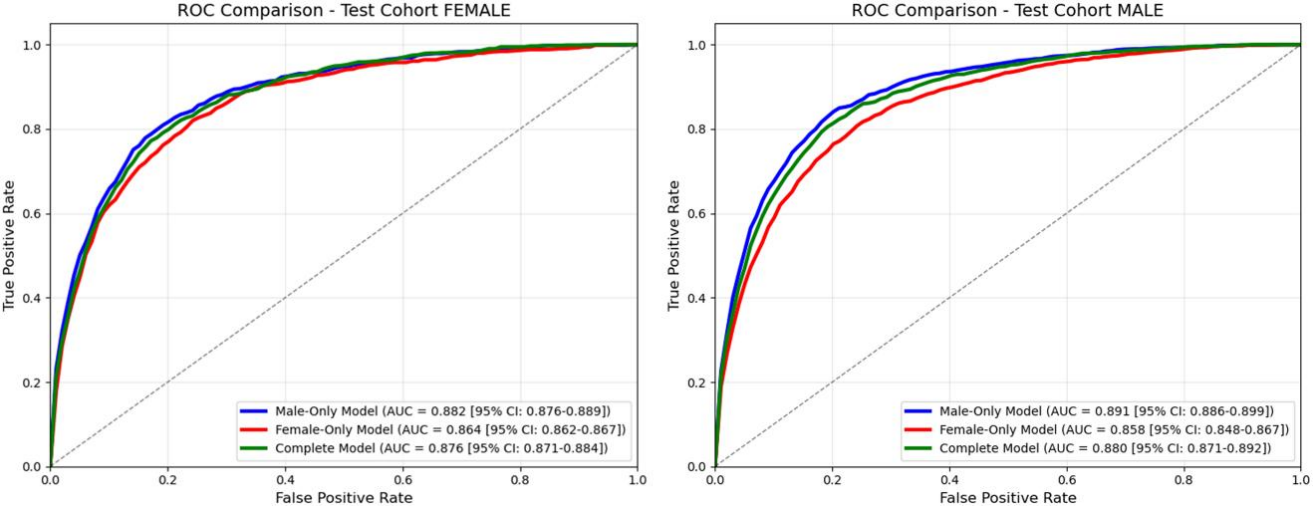
ECGFM AUROC curves for LVSD (EF < 40%). (*A*, Left panel) AUROC plot for models trained on different cohorts, (Complete Dataset, Male only, Female only) and tested on the Female only dataset. (*B*, Right panel) AUROC curves for models trained on different cohorts and tested on the Male only dataset.

**Figure 3:**
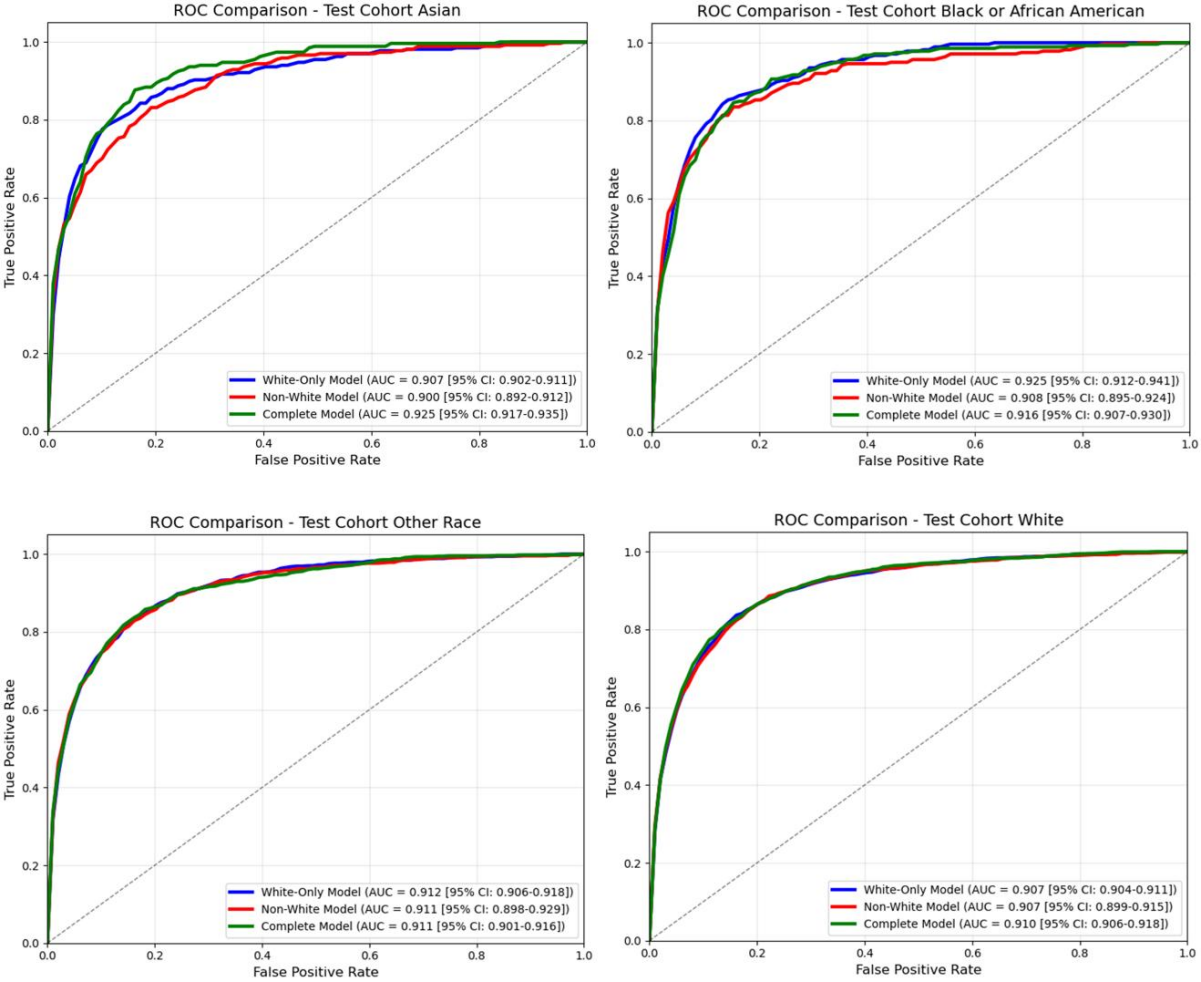
(*A–D*) Precision-recall curves for models trained on complete, hite-only, and non-White cohorts and tested on Asian only, lack/African American only, other race, and White datasets. (*E–F*) Precision-recall curves for models trained on complete, Male-only, and Female-only cohorts and tested on Female-only and Male-only datasets.

The ECGFM for predicting LVSD demonstrated consistent performance across all sex-based configurations (***Table 2***). The ECG-FM tested on the female-only set had an AUROC of 0.882 (95% CI: 0.876–0.889) when trained in a male-only configuration with an area under the precision-recall curve (AUPRC) of 0.452 (95% CI: 0.435–0.475) and an AUROC of 0.864 (95% CI: 0.862–0.867) when trained on a female-only dataset with an AUPRC 0.409 (95% CI: 0.400–0.415) (*Figures 2A* and *3E*). The ECG-FM tested on a male-only dataset had an AUROC of 0.891 (95% CI: 0.886–0.899) when trained in a male-only configuration with an AUPRC of 0.623 (95% CI: 0.620–0.626) and an AUROC of 0.858 (95% CI: 0.848–0.867) in a female-only configuration with an AUPRC of 0.558 (95% CI: 0.549–0.563) (*Figures 2B* and *3F*). The AUROC was 0.875 (95% CI: 0.871–0.884) for the female test dataset with an AUPRC 0.433 (95% CI: 0.402–0.457) and 0.880 (95% CI: 0.871–0.892) for the male test dataset with an AUPRC 0.596 (95% CI: 0.582–0.607) when trained on the complete sex subgroup. (*Figures 2* and *3E-F*)

**Table 2:** Model performance for LVSD from ECG-FM with sensitivity, specificity, PPV, NPV, and accuracy

Dataset	Model	Sensitivity	Specificity	PPV	NPV	Accuracy
Complete	White-Only Model	0.832	0.846	0.405	0.976	0.845
Complete	Non-White Model	0.834	0.832	0.387	0.976	0.832
Complete	Complete Model	0.844	0.835	0.392	0.977	0.836
White	White-Only Model	0.852	0.828	0.379	0.979	0.831
White	Non-White Model	0.857	0.813	0.361	0.979	0.818
White	Complete Model	0.846	0.828	0.38	0.978	0.83
Other race	White-Only Model	0.842	0.835	0.395	0.977	0.836
Other race	Non-White Model	0.857	0.822	0.387	0.979	0.826
Other race	Complete Model	0.832	0.849	0.411	0.976	0.847
Asian	White-Only Model	0.85	0.843	0.377	0.982	0.843
Asian	Non-White Model	0.82	0.831	0.342	0.978	0.83
Asian	Complete Model	0.858	0.865	0.415	0.983	0.865
Black or African American	White-Only Model	0.864	0.857	0.55	0.969	0.858
Black or African American	Non-White Model	0.835	0.857	0.54	0.963	0.854
Black or African American	Complete Model	0.882	0.827	0.509	0.972	0.836

Dataset	Model	Sensitivity	Specificity	PPV	NPV	Accuracy
Complete	Male-Only Model	0.831	0.804	0.349	0.974	0.807
Complete	Female-Only Model	0.806	0.769	0.306	0.969	0.773
Complete	Complete Model	0.81	0.808	0.347	0.971	0.808
MALE	Male-Only Model	0.836	0.807	0.429	0.966	0.811
MALE	Female-Only Model	0.805	0.765	0.374	0.958	0.771
MALE	Complete Model	0.816	0.801	0.416	0.962	0.803
FEMALE	Male-Only Model	0.81	0.815	0.258	0.982	0.815
FEMALE	Female-Only Model	0.784	0.801	0.244	0.979	0.799
FEMALE	Complete Model	0.816	0.791	0.24	0.982	0.793

**Figure 4:**
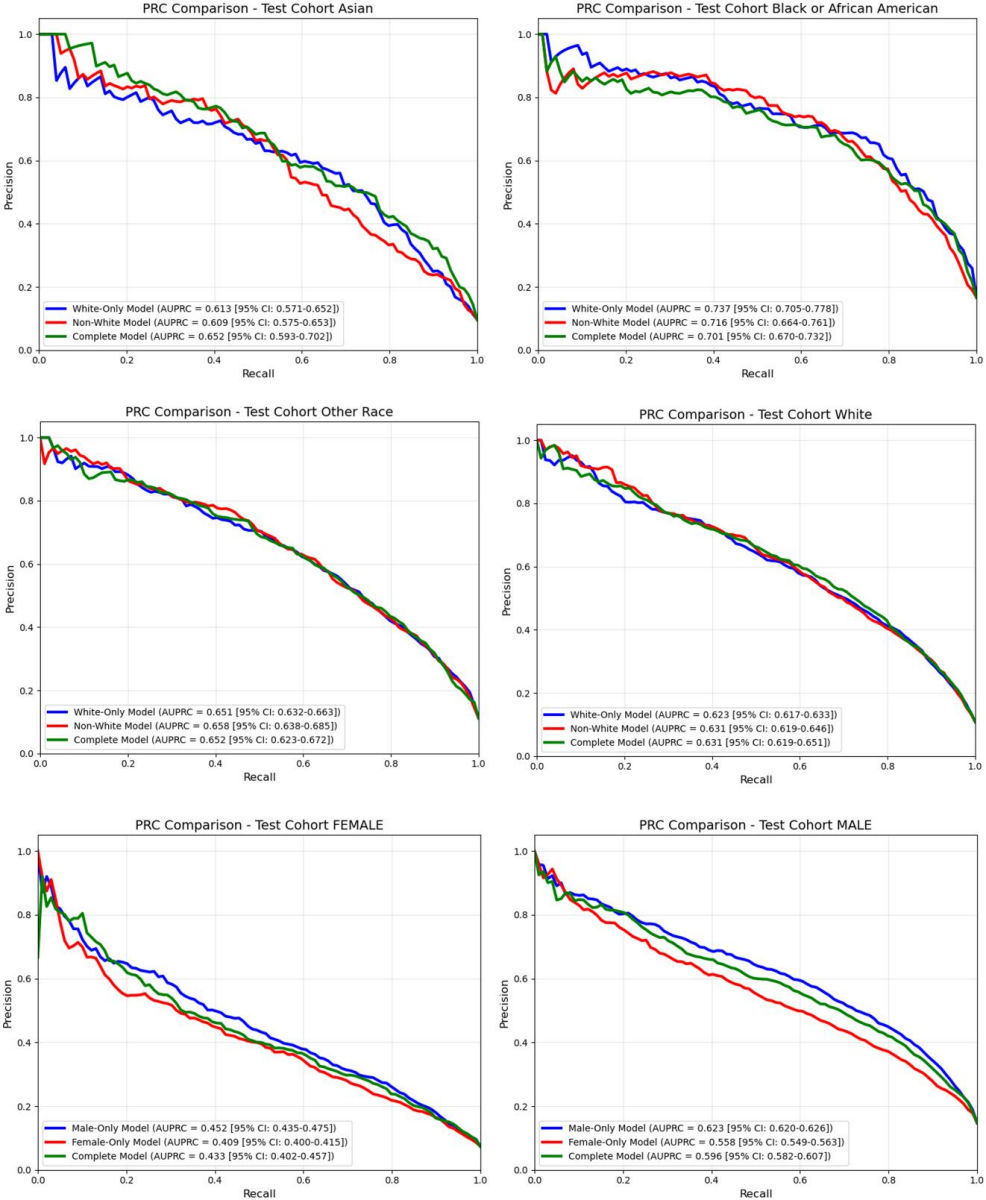
(*A*) AUROC curves for models trained on different cohorts, on Asian only dataset. (*B*) AUROC curves for models trained on different cohorts (Complete Dataset, White only, Complete except White) on Black or African only dataset. (*C*) AUROC curves for models trained on different cohorts, on other races only dataset. (*D*) AUROC curves for models trained on different cohorts on White only dataset.

Similarly robust performance was observed across all race-based configurations. The model on a Black or African American-only test set yielded a high AUROC and similar AUPRCs irrespective of which training subgroup was used: AUROC 0.925 (95% CI: 0.912–0.941) and AUPRC 0.737 (95% CI: 0.705–0.778) for White-only configuration, AUROC 0.908 (95% CI: 0.895–0.924) and AUPRC 0.716 (95% CI: 0.664–0.761) for all non-White configuration, and AUROC 0.916 (95% CI: 0.907–0.930) and AUPRC 0.701 (95% CI: 0.670–0.732) with the complete dataset (*Figures 4A* and *3B*). Similarly, on an Asian test dataset the ECGFM achieved an AUROC of 0.907 (95% CI: 0.902–0.911) and AUPRC 0.613 (95% CI: 0.571–0.652) trained on a White-only dataset, AUROC 0.900 (95% CI: 0.892–0.912) and AUPRC 0.609 (95% CI: 0.575–0.653) on an all non-White set, and AUROC 0.925 (95% CI: 0.917–0.935) and AUPRC 0.652 (95% CI: 0.593–0.702) using the complete dataset (*Figures 4B* and *3A*). The Other testing dataset also showed excellent performance, with an AUROC of 0.912 (95% CI: 0.906–0.918) and AUPRC 0.651 (95% CI: 0.632–0.663) trained by the White-only configuration, AUROC 0.911 (95% CI: 0.898–0.929), and AUPRC 0.658 (95% CI: 0.638–0.685) using a non-White training configuration, and AUROC 0.911 (95% CI: 0.901–0.916) and AUPRC 0.652 (95% CI: 0.623–0.672) with the complete dataset (*Figures 4C* and *3C*). Finally, the model tested on the White-only patients only data achieved an AUROC of 0.907 (95% CI: 0.904–0.911) and AUPRC 0.623 (95% CI: 0.617–0.663) using the White-only configuration, AUROC 0.907 (95% CI: 0.899–0.915) and AUPRC 0.631 (95% CI: 0.619–0.646) trained on an all non-White dataset, and AUROC 0.910 (95% CI: 0.906–0.918) and AUPRC 0.631 (95% CI: 0.619–0.651) with the complete dataset (*Figures 4D* and *3D*). Again, we compared these results to a demographically balanced foundational model training configuration, which demonstrated an overall AUROC across all groups of 0.91 (95% CI: 0.913–0.914) (*Figure 4*).

**Figure 5:**
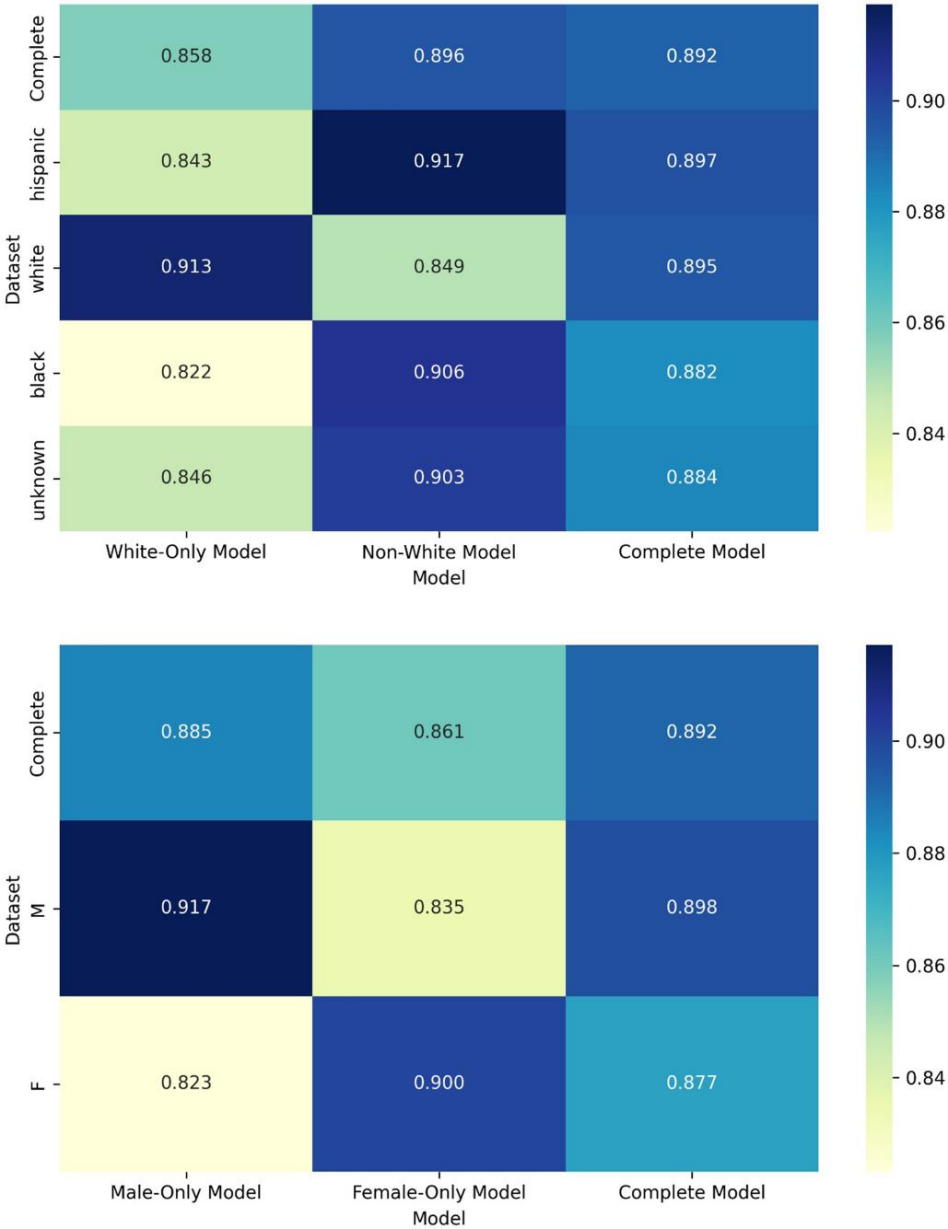
External validation of ECGFM on EchoNext cohort. (Top) Heatmap of AUROC scores for all sex-based configurations. (Bottom) Heatmap of AUROC scores for all race-based configurations.

Next, we evaluated model performance in an external cohort, the publicly available EchoNext benchmark, as an independent test set. Compared with the internal cohort, baseline demographics consisted of 29 985 male patients (54.9%) and 24 651 female patients (45.1%), as well as 16 044 (29.4%) White, 9114 (16.7%) Black, 16 341 (30.0%) Hispanic, 7035 Other (12.9%) patients, and 6102 (11.2%) Unknown. The external cohort clinical contexts included emergency (28.5%), inpatient (52.1%), outpatient (15.8%), and procedural settings (3.60%). The ECGFM for predicting LVSD showed preserved performance with AUROCs of 0.823–0.917 across sex-based configurations and AUROCs of 0.822–0.917 across race-based configurations (*Figure 5*).

## Discussion

In this study, we introduce an ECG foundational model for the diagnosis of LVSD and evaluate the impact of demographic composition in training sets on its performance. By comparison to previous deep learning approaches, foundational models potentially offer broad generalizability and address persistent concerns surrounding fairness and equity in clinical applications. Here we show an ECGFM which demonstrates robust generalization across sex and racial groups, indicating that diagnostic features for predicting LVSD may be invariant to demographic variations. With the right approach, AI health models can be tools that support equity by reducing the risk of algorithmic bias.

### Comparison with other studies

Our results are noteworthy when considered in the context of prior literature. Most previous AI-ECG studies have relied on convolutional neural networks and training datasets that are relatively limited in size and diversity. These models have occasionally demonstrated reduced generalizability when applied to underrepresented populations, and a head-to-head comparison study was hindered by the limited availability of these models.^27–29^ In contrast, our model leverages transfer learning and self-supervised pretraining strategies, which are less susceptible to overfitting demographic-specific patterns. Instead, foundational models trained on large and diverse (even if unbalanced) datasets may achieve both high accuracy and equity, provided that downstream validation includes diverse populations. Other efforts using foundational models highlight their multi-task generalizability in screening for structural heart disease (up to 26 subtypes), single and multi-lead ECGs, and impaired myocardial flow reserve.^30–33^ This has important implications for AI deployment in resource-limited settings where collecting balanced datasets is impractical. By demonstrating broad demographic generalizability, our work lowers barriers for implementing AI-driven ECG screening, especially in regions or health systems with limited data availability.

#### Clinical implications

The need for inexpensive and effective screening for early LVSD is of paramount significance. Early diagnosis of reduced LV systolic function is critical in guiding heart failure therapies, and isolated asymptomatic LVSD itself has prognostic value, being a harbinger of incident or progressive HF and mortality.^34,35^ The economic burden of HF is substantial, with estimated direct and indirect costs worldwide (in 197 countries) totalling $108 billion,^36^ much of this resulting from readmissions for HF and inpatient services.^37^ Thus, early detection and prevention of clinical HF can be of critical importance to reduce healthcare costs. AI-ECG approaches compare favourably to biomarker-based strategies; utilization of NT-proBNP for screening of systolic dysfunction demonstrated an AUROC of 0.70.^38^ The ECG represents a standardized, universally available, and inexpensive tool adaptable to many resource-limited settings and potentially compatible with wearables and smartphones. Expansion of the ECG’s clinical utility is key to allowing patients access to effective guideline-directed medicines and advanced heart failure therapies (e.g. cardiac implantable devices).

Our ECGFM leverages a Vision Transformer (ViT) architecture pretrained through self-supervised learning on approximately one million diverse ECG signals, enabling robust and generalizable feature extraction. Unlike traditional convolutional neural networks used in prior AI-ECG methods, which primarily capture local temporal patterns, ECGFM efficiently encodes both temporal and spatial dependencies inherent in ECG signals. Consequently, ECGFM demonstrated consistently high diagnostic performance (AUROC ≥ 0.85) across demographic subgroups, including sex (male and female) and race (White, Asian, Black, Hispanic/Latino, and American Indian/Native Alaskan), even when trained on demographically imbalanced datasets. It should be noted that our cohort was comprised of patients receiving clinical care and therefore caution should be exercised when extrapolating to other use cases (e.g. asymptomatic screening), which would require prospective validation. These findings suggest that, given sufficient scale, ECG-based predictions for cardiac conditions such as reduced LVEF can maintain equitable and robust performance independent of unequal demographic representation in training data.

### Strengths and limitations

The demonstrated demographic generalizability of the ECGFM model for LVSD has important implications for AI-ECG diagnostic technology, especially in low-resource settings.^13,39^ It should be noted that all data sources were US-based, and therefore, this is a limitation for generalizability, particularly without external validation. Our overall study cohort was potentially enriched for patients already undergoing evaluation for cardiac disease due to the requirement for both an ECG and TTE within 30 days, which may explain relatively high rates of atrial fibrillation (32%) and ventricular tachycardia/fibrillation (∼10%). By achieving robust generalization despite demographic imbalances with at least equal performance for predicting LVSD, ECGFM substantially lowers the logistical and economic barriers traditionally associated with assembling large, balanced training datasets. This capability is particularly advantageous for deploying AI-driven diagnostics in underserved regions or addressing conditions with low prevalence, where comprehensive demographic representation is often impractical or economically unfeasible. Furthermore, our results carry significant policy implications for AI equity, suggesting that regulatory guidelines emphasizing strict demographic balancing may be usefully supplemented or reconsidered.

However, our results should not be interpreted to mean that fairness considerations can be ignored. While AUROC parity across subgroups is reassuring, other fairness dimensions such as calibration, false positive/negative disparities, and potential sociotechnical biases remain critical. Moreover, AI models can encode structural inequities from their healthcare environment (e.g. access to care, comorbidity burden), which cannot be fully addressed by model architecture alone.^13,39^ Although promising, at this time the results of our study should not be generalized to other AI-enabled tasks (prediction of structural cardiac dimensions, atrial fibrillation, etc.) until formally studied for demographic-dependent features. These issues may be even more salient for the application of AI models to tasks other than LVSD. Therefore, we advocate for continuous demographic monitoring of AI models in clinical deployment, especially in high-stakes applications such as heart failure screening.

## Conclusion

A transformer-based ECG foundational model pretrained on diverse datasets detects LVSD with high accuracy and demographic parity, even when trained on imbalanced cohorts. These findings support the scalability and equitable deployment of foundational AI models in cardiovascular screening and highlight the importance of continued fairness monitoring and external validation.

## References

[1] Collaborators G 2017 D and II and P . Global, regional, and national incidence, prevalence, and years lived with disability for 354 diseases and injuries for 195 countries and territories, 1990-2017: a systematic analysis for the Global Burden of Disease Study 2017. Lancet 2018;392:1789–1858. 30496104 10.1016/S0140-6736(18)32279-7 PMC6227754

[2] Savarese G, Becher PM, Lund LH, Seferovic P, Rosano GMC, Coats AJS. Global burden of heart failure: a comprehensive and updated review of epidemiology. Cardiovasc Res 2023;118:3272–3287. 35150240 10.1093/cvr/cvac013

[3] Heidenreich PA, Bozkurt B, Aguilar D, Allen LA, Byun JJ, Colvin MM, et al 2022 AHA/ACC/HFSA guideline for the management of heart failure: a report of the American College of Cardiology/American Heart Association Joint Committee on Clinical Practice Guidelines. Circulation 2022;145:e895–e1032. 35363499 10.1161/CIR.0000000000001063

[4] Lee E, Ito S, Miranda WR, Lopez-Jimenez F, Kane GC, Asirvatham SJ, et al Artificial intelligence-enabled ECG for left ventricular diastolic function and filling pressure. npj Digit Med 2024;7:1–7. 38182738 10.1038/s41746-023-00993-7 PMC10770308

[5] Siontis KC, Abreau S, Attia ZI, Barrios JP, Dewland TA, Agarwal P, et al Patient-level artificial intelligence–enhanced electrocardiography in hypertrophic cardiomyopathy. JACC Adv 2023;2:100582. 38076758 10.1016/j.jacadv.2023.100582 PMC10702858

[6] Adedinsewo DA, Morales-Lara AC, Afolabi BB, Kushimo OA, Mbakwem AC, Ibiyemi KF, et al Artificial intelligence guided screening for cardiomyopathies in an obstetric population: a pragmatic randomized clinical trial. Nat Med 2024;30:2897–2906. 39223284 10.1038/s41591-024-03243-9 PMC11485252

[7] Yao X, Rushlow DR, Inselman JW, McCoy RG, Thacher TD, Behnken EM, et al Artificial intelligence–enabled electrocardiograms for identification of patients with low ejection fraction: a pragmatic, randomized clinical trial. Nat Med 2021;27:815–819. 33958795 10.1038/s41591-021-01335-4

[8] Amadio JM, Grogan M, Muchtar E, Lopez-Jimenez F, Attia ZI, AbouEzzeddine O, et al Predictors of mortality by an artificial intelligence enhanced electrocardiogram model for cardiac amyloidosis. ESC Heart Fail 2025;12:677–682. 39215684 10.1002/ehf2.15061 PMC11769637

[9] Thao V, Zhu Y, Tseng AS, Inselman JW, Borah BJ, McCoy RG, et al Cost-effectiveness of artificial intelligence-enabled electrocardiograms for early detection of low ejection fraction: a secondary analysis of the electrocardiogram artificial intelligence-guided screening for low ejection fraction trial. Mayo Clin Proc Digit Health 2024;2:620–631. 40206537 10.1016/j.mcpdig.2024.10.001 PMC11975989

[10] Bazoukis G, Hall J, Loscalzo J, Antman EM, Fuster V, Armoundas AA. The inclusion of augmented intelligence in medicine: a framework for successful implementation. CR Med 2022;3:100485. 10.1016/j.xcrm.2021.100485 PMC8784713 35106506

[11] Armoundas AA, Narayan SM, Arnett DK, Spector-Bagdady K, Bennett DA, Celi LA, et al Use of artificial intelligence in improving outcomes in heart disease: a scientific statement from the American Heart Association. Circulation 2024;149:e1028–e1050. 38415358 10.1161/CIR.0000000000001201 PMC11042786

[12] Zheng J, Ani C, Abudayyeh I, Zheng Y, Rakovski C, Yaghmaei E, et al A review of racial differences and disparities in ECG. Int J Environ Res Public Health 2025;22:337. 40238300 10.3390/ijerph22030337 PMC11942291

[13] Bollepalli SC, Isselbacher EM, Singh JP, Armoundas AA. Non-genetic factors determine deep learning identified ECG differences between Black and White healthy subjects. npj Cardiovasc Health 2025;2:51. 41080706 10.1038/s44325-025-00087-1 PMC12513828

[14] Pearson TA, Vitalis D, Pratt C, Campo R, Armoundas AA, Au D, et al The science of precision prevention. JACC Adv 2024;3:100759. 38375059 10.1016/j.jacadv.2023.100759 PMC10876066

[15] Larrazabal AJ, Nieto N, Peterson V, Milone DH, Ferrante E. Gender imbalance in medical imaging datasets produces biased classifiers for computer-aided diagnosis. Proc Natl Acad Sci U S A 2020;117:12592–12594. 32457147 10.1073/pnas.1919012117 PMC7293650

[16] Vyas DA, Eisenstein LG, Jones DS. Hidden in plain sight—reconsidering the use of race correction in clinical algorithms. N Engl J Med 2020;383:874–882. 32853499 10.1056/NEJMms2004740

[17] Obermeyer Z, Powers B, Vogeli C, Mullainathan S. Dissecting racial bias in an algorithm used to manage the health of populations. Science 2019;366:447–453. 31649194 10.1126/science.aax2342

[18] Moor M, Banerjee O, Abad ZSH, Krumholz HM, Leskovec J, Topol EJ, et al Foundation models for generalist medical artificial intelligence. Nature 2023;616:259–265. 37045921 10.1038/s41586-023-05881-4

[19] Christensen M, Vukadinovic M, Yuan N, Ouyang D. Vision–language foundation model for echocardiogram interpretation. Nat Med 2024;30:1481–1488. 38689062 10.1038/s41591-024-02959-y PMC11108770

[20] McKeen K, Oliva L, Masood S, Toma A, Rubin B, Wang B. ECG-FM: an open electrocardiogram foundation model. JAMIA Open 2025;8:ooaf122. 41113504 10.1093/jamiaopen/ooaf122 PMC12530324

[21] Yu H, Guo P, Sano A. ECG semantic integrator (ESI): a foundation ECG model pretrained with LLM-enhanced cardiological text. Transact Mach Learn Res 2024;2024:2818. 41816367 PMC12974696

[22] Heidenreich PA, Bozkurt B, Aguilar D, Allen LA, Byun JJ, Colvin MM, et al 2022 AHA/ACC/HFSA guideline for the management of heart failure. JACC 2022;79:e263–e421. 35379503 10.1016/j.jacc.2021.12.012

[23] Elias P, Finer J. EchoNext: a dataset for detecting echocardiogram-confirmed structural heart disease from ECGs. PhysioNet 2025.

[24] Goldberger AL, Amaral LAN, Glass L, Hausdorff JM, Ivanov P, Mark RG, et al PhysioBank, PhysioToolkit, and PhysioNet: components of a new research resource for complex physiologic signals. Circulation 2000;101:e215–e220. 10851218 10.1161/01.cir.101.23.e215

[25] He K, Chen X, Xie S, Li Y, Dollár P, Girshick R. In: *Proceedings of the IEEE/CVF conference on computer vision and pattern recognition*, 2022, p16000–16009.

[26] Yang S, Lian C, Zeng Z. Masked Autoencoder for ECG Representation Learning. In: 2022 12th International Conference on Information Science and Technology (ICIST) [Internet]. 2022 [cited 2025 Aug 10]. p. 95–98. Available from: https://ieeexplore.ieee.org/abstract/document/9926900

[27] Khan S, Qayyum K, Qadeer A, Khalid M, Anthony S, Khan W, et al Efficacy of AI models in detecting heart failure using ECG data: a systematic review and meta-analysis. Cureus 2025;17:e78683. 10.7759/cureus.78683 PMC11891813 40065863

[28] Yagi R, Goto S, Katsumata Y, MacRae CA, Deo RC. Importance of external validation and subgroup analysis of artificial intelligence in the detection of low ejection fraction from electrocardiograms. Eur Heart J Digit Health 2022;3:654–657. 36710903 10.1093/ehjdh/ztac065 PMC9779862

[29] Croon PM, Boonstra MJ, Allaart CP, Arends BKO, Dhingra LS, Huang YC, et al Artificial intelligence–enhanced electrocardiogram models for detection of left ventricular dysfunction. JACC Adv 2026;5:102572. 41564731 10.1016/j.jacadv.2025.102572 PMC12856472

[30] Oikonomou EK, Batinica B, Dhingra LS, Aminorroaya A, Coppi A, Khera R. TARGET-AI: a foundational approach for the targeted deployment of artificial intelligence electrocardiography in the electronic health record. NEJM AI 2026;3:AIoa2500588.

[31] Li J, Aguirre AD, Junior VM, Jin J, Liu C, Zhong L, et al An electrocardiogram foundation model built on over 10 million recordings. NEJM AI 2025;2:AIoa2401033. 10.1056/aioa2401033 PMC12327759 40771651

[32] Nolin-Lapalme A, Sowa A, Delfrate J, Tastet O, Corbin D, Kulbay M, et al Foundation models for electrocardiogram interpretation: clinical implications. Eur Heart J 2026;47:2174–2186. 41568699 10.1093/eurheartj/ehaf1119 PMC13178678

[33] Moody JB, Poitrasson-Rivière A, Renaud JM, Hagio T, Alahdab F, Al-Mallah MH, et al A foundation transformer model with self-supervised learning for ECG-based assessment of cardiac and coronary function. NEJM AI 2025;2:AIoa2500164. 10.1056/aioa2500164 PMC12724683 41446031

[34] Pandhi J, Gottdiener JS, Bartz TM, Kop WJ, Mehra MR. Comparison of characteristics and outcomes of asymptomatic versus symptomatic left ventricular dysfunction in subjects 65 years old or older (from the Cardiovascular Health Study). Am J Cardiol 2011;107:1667–1674. 21575752 10.1016/j.amjcard.2011.01.051 PMC4143416

[35] Wang TJ, Evans JC, Benjamin EJ, Levy D, LeRoy EC, Vasan RS. Natural history of asymptomatic left ventricular systolic dysfunction in the community. Circulation 2003;108:977–982. 12912813 10.1161/01.CIR.0000085166.44904.79

[36] Cook C, Cole G, Asaria P, Jabbour R, Francis DP. The annual global economic burden of heart failure. Int J Cardiol 2014;171:368–376. 24398230 10.1016/j.ijcard.2013.12.028

[37] Ogah OS, Stewart S, Onwujekwe OE, Falase AO, Adebayo SO, Olunuga T, et al Economic burden of heart failure: investigating outpatient and inpatient costs in Abeokuta, Southwest Nigeria. PLoS One 2014;9:e113032. 25415310 10.1371/journal.pone.0113032 PMC4240551

[38] Bhalla V, Isakson S, Bhalla MA, Lin JP, Clopton P, Gardetto N, et al Diagnostic ability of B-type natriuretic peptide and impedance cardiography: testing to identify left ventricular dysfunction in hypertensive patients. Am J Hypertens 2005;18:73S–81S. 15752936 10.1016/j.amjhyper.2004.11.044

[39] Bazoukis G, Loscalzo J, Hall JL, Bollepalli SC, Singh JP, Armoundas AA. Impact of social determinants of health on cardiovascular disease. J Am Heart Assoc 2025;14:e039031. 40035388 10.1161/JAHA.124.039031 PMC12132660

